# Biologics for severe, chronic plaque psoriasis: An Australian cost-utility analysis

**DOI:** 10.1016/j.jdin.2021.06.004

**Published:** 2021-08-09

**Authors:** Helen Y. Sun, Elena Keller, Harish Suresh, Deshan F. Sebaratnam

**Affiliations:** a Faculty of Medicine and Health, The University of New South Wales, Kensington, New South Wales, Australia; b Department of Dermatology, Liverpool Hospital, Liverpool, New South Wales, Australia; c Centre for Big Data Research in Health, The University of New South Wales, Kensington, New South Wales, Australia; d Faculty of Business, The University of New South Wales, Kensington, New South Wales, Australia

**Keywords:** adalimumab, Australia, biologic therapy, cost-effectiveness analysis, cost-utility analysis, cost-benefit analysis, cyclosporine, dermatologists, etanercept, guselkumab, health economics, infliximab, ixekizumab, psoriasis, quality of life, risankizumab, tildrakizumab, AUD, Australian dollar, BSC, best supportive care, CI, confidence interval, ICUR, incremental cost-utility ratio, IL, interleukin, QALY, quality-adjusted life years, PASI, Psoriasis Area and Severity Index, PASI75, 75% improvement from the initial Psoriasis Area and Severity Index Score, PBS, Pharmaceutical Benefits Scheme, RCT, randomized-controlled trial, TNF-α, tumor necrosis alpha, WTP, willingness-to-pay

## Abstract

**Background:**

Biologics are a good therapeutic option for severe, chronic plaque psoriasis; however, they come with significant cost to the health care system.

**Objective:**

To conduct a cost-utility analysis of outpatient biologics (adalimumab, etanercept, guselkumab, ixekizumab, risankizumab, secukinumab, tildrakizumab, and ustekinumab) available to adults with severe, chronic plaque psoriasis from the perspective of the Australian health care system.

**Methods:**

A Markov cohort model was constructed to estimate the quality-adjusted life years (QALYs) and costs accrued for treatment pathways commencing with different first-line biologics, over a 96-week time horizon. The model adhered to the Australian Pharmaceutical Benefits Scheme eligibility criteria and guidelines.

**Results:**

A biologic treatment pathway commencing on tildrakizumab was the most cost-effective first-line treatment (Australian dollar 39,930; total utility of 1.57 QALYs over 96 weeks). First-line secukinumab and risankizumab had incremental cost-utility ratios of Australian dollar 194,524/QALY and Australian dollar 479,834/QALY, respectively, when compared with first-line tildrakizumab.

**Limitations:**

The efficacy and utility input parameters were derived from international randomized control trials and patients from the United Kingdom, respectively. Findings from this study cannot be generalized beyond Australia.

**Conclusion:**

Tildrakizumab may be considered as first-line treatment for adult patients with severe, chronic plaque psoriasis embarking on biologic therapy, from the economic perspective of the Australian health care system.

**Capsule Summary**

• Biologics are a good therapeutic option for severe, chronic plaque psoriasis; however, they come with significant cost to the health care system.
• This cost-utility study supports tildrakizumab as first-line treatment for adults with chronic plaque psoriasis embarking on biologic therapy in the Australian setting.

## Introduction

In the last decade, the management of severe psoriasis has been revolutionized by the advent of biologics. Biologics generally have a more favorable side effect profile compared with those of conventional systemic therapies such as acitretin, methotrexate, or cyclosporine.^1^ However, biologics are associated with significant acquisition costs. In Australia, the biologics available for psoriasis are the tumor necrosis factor-alpha (TNF-α) inhibitors: adalimumab, infliximab, and etanercept; the interleukin (IL)-12/23 inhibitor: ustekinumab; the IL-17 inhibitors: secukinumab and ixekizumab; and the IL-23 inhibitors: guselkumab, risankizumab, and tildrakizumab.^2^ To facilitate access, biologics are subsidized by the Australian Pharmaceutical Benefits Scheme (PBS). The maximum patient co-payment for each dose of biologic is 41.00 Australian dollars (AUD) or AUD6.60 for concession holders^3^—the remaining cost is paid by the government (Table I).^4^, ^5^, ^6^, ^7^, ^8^, ^9^, ^10^, ^11^, ^12^ Because psoriasis is incurable, patients require long-term treatment, and the financial burden of this on the health care system is considerable.

**Table I:** Probabilities of PASI75 based on meta-analyses of randomized control trials and costs of biologics per dispense in Australia, as of 18 September 2020

Generic name of biologic (dosage)	Dispensed price for maximum quantity∗ (AUD)	Cost to health care system per dispense† (AUD)	Dosing frequency	Probability of PASI75 at the end of the induction period (% [95% CI])	Probability of PASI75 at the end of maintenance periods (% [95% CI])
Adalimumab (40 mg)	1173^4^	1132	Week 0, 1, and every 2 weeks thereafter	69.5 (66.0-72.6)^1^	67.1 (52.9-78.7)^1^
Etanercept (50 mg)	1067^5^	1026	Weekly	40.1 (35.4-45.1)^1^	55.5 (50.1-60.9)^1^
Guselkumab (100 mg)	3812^6^	3771	Week 0, 4, and every 8 weeks thereafter	86.8 (83.8-89.4)^1^	88.2 (84.6-91.1)^1^
Ixekizumab (80 mg)	3437^7^	3396	Week 0, 2, 4, 6, 8, 10, 12, and every 4 weeks thereafter	88.8 (86.5-90.9)^1^	85.0 (79.2-89.4)^1^
Risankizumab (150 mg)	5416^8^	5375	Week 0, 4, and every 12 weeks thereafter	89.2 (86.9-91.3)^1^	90.1 (86.3-92.9)^1^
Secukinumab (300 mg)	1481^9^	1440	Week 0, 1, 2, 3, 4, and every 4 weeks thereafter	83.1 (80.2-85.7)^1^	88.6 (80.6-93.6)^1^
Tildrakizumab (100 mg)	3287^10^	3246	Week 0, 4, and every 12 weeks thereafter	62.9 (57.3-68.4)^1^	87.8 (84.3-91.4)^11^
Ustekinumab (45 mg ≤100 kg, 90 mg >100 kg)	3951^12^	3910	Week 0, 4, and every 12 weeks thereafter	69.7 (66.3-73.1)^1^	72.5 (65.9-78.2)^1^

*AUD*, Australian dollar; *PASI75*, 75% improvement from the initial Psoriasis Area and Severity Index Score.

∗ The dispensed price for maximum quantity is the maximum price that the Australian government will pay for a drug, incorporating price premiums, all fees, mark-ups, and patient contributions.

† Assuming these patients were not concession card holders.

There exists no published literature on the cost-effectiveness of the biologics for psoriasis available through the Australian PBS. Herein, this study uses the latest efficacy and cost data to investigate the health and economic outcomes of the biologic options available to Australian patients.

## Methods

A cost-utility analysis was conducted to compare adalimumab, etanercept, guselkumab, ixekizumab, risankizumab, secukinumab, tildrakizumab, and ustekinumab as first-line treatments for adults with severe, chronic plaque psoriasis. The outcomes included total cost (measured in 2020 AUD), total quality-adjusted life years (QALYs) accrued, and incremental cost-utility ratios (ICURs). The University of New South Wales granted this study ethics approval (HC190297). This economic evaluation follows the Consolidated Health Economic Evaluation Reporting Standards checklist for reporting economic evaluations.

### Criterion for starting biologic therapy

The PBS requires patients to have severe psoriasis to start biologic therapy. Severe psoriasis is defined quantitatively as a Psoriasis Area and Severity Index (PASI) score >15 for at least 6 months.^13^ Patients must also have failed to achieve an adequate response following a minimum of 6 weeks of treatment with at least 2 of the following 5 treatments: phototherapy, methotrexate, apremilast, cyclosporine, or acitretin.^13^

### Model design

**Fig 1:**
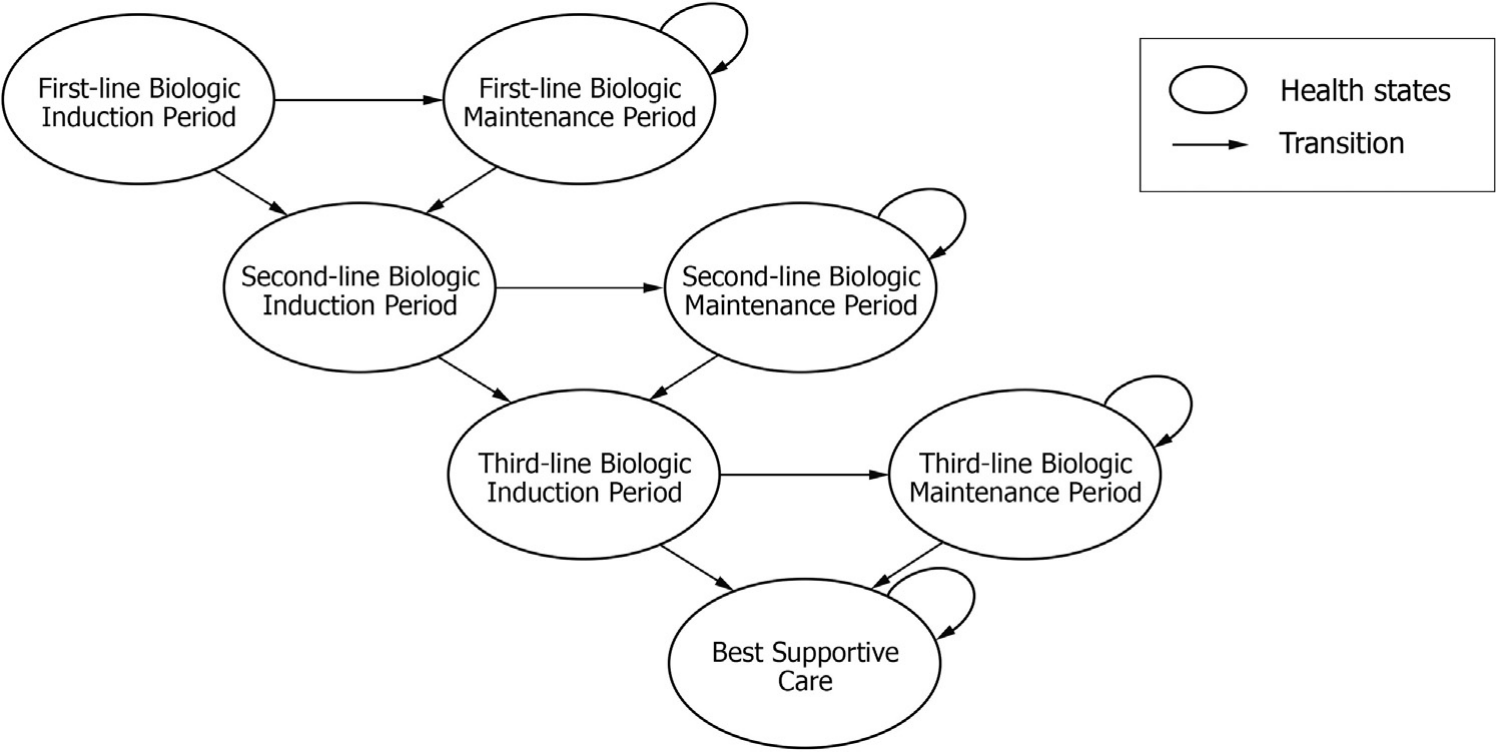
State transition model of one arm of the Markov Model.

A Markov Model was designed with TreeAge Pro Software (Version 2020 R1.1). The model had 8 treatment arms, each commencing on a unique first-line biologic. The state transition model of 1 arm of the Markov Model can be visualized in Fig 1, and a more detailed version can be found in Supplemental Figure 1 (available via Mendeley at https://data.mendeley.com/datasets/79b2ns555y/1). The model utilized a cycle-length of 12 weeks, allowing for 8 Markov cycles over the 96-week time horizon. This corresponds to PBS regulations requiring that treatment with each drug begins with a 12-week induction period followed by 24-week maintenance periods. Under PBS regulations, patients are required to be reviewed by dermatologists for their achievement of 75% improvement in PASI score (PASI75) at the end of each period. Patients who did not achieve PASI75 were considered to have failed treatment and were transitioned to second and third-line biologic options. Patients who failed third-line biologic therapy were transitioned to best supportive care (BSC). Given the short time horizon of 96 weeks, discounting was not applied to this model.

### Model assumptions

The model assumptions were as follows:

1. Patients who failed to achieve PASI75 were, on average, assumed to fail halfway through a 12-week cycle. Within-cycle correction was used to apply this assumption.
2. Risankizumab and ixekizumab were used as second- and third-line treatments, respectively, as these were the treatments with the highest and second-highest efficacy in the induction period.^2^ This assumption was made to maximize the probability of remaining on the biologic. In the case of patients starting on either risankizumab or ixekizumab, the other biologic was used as the second-line treatment. Patients then transitioned to guselkumab as a third-line treatment as this was the third most effective drug in the induction period.^2^
3. The transition probabilities for every 24-week review were assumed to be the same because of limited data in the literature.
4. Patients receiving BSC after failing to achieve PASI75 with 3 trials of biologics had zero chance of achieving PASI75 thereafter.
5. Subcutaneous biologics were self-administered, reflecting current clinical practice in Australia.
6. Psoriasis had no effect on natural mortality.^14^

### Clinical data inputs

The transition probabilities in the Markov Model were derived from Armstrong et al's^2^ systematic review and meta-analysis, which synthesized data from 60 phase II, III, or IV randomized controlled trials (RCTs) of biologics. The PASI75 response rates were used as the measure for biologic efficacy. The short-term induction period efficacy (12-16 weeks) from the network meta-analysis was used for the induction period transition probabilities, and long-term efficacy data (44-60 weeks) from the traditional random effects meta-analysis were used for all subsequent 24-week maintenance period transition probabilities (Table I). Long-term trial data (52 weeks) for tildrakizumab were derived directly from Phase III trials.^11^

### Cost inputs

This analysis was performed from the perspective of the Australian health care system and only considered direct medical costs. These included biologic acquisition costs factoring in a patient co-payment of AUD41.00, BSC costs, and monitoring costs (including medical visits and investigations) (Tables I and II).^15^ All costs were obtained from publicly available information from the PBS and reported in 2020 AUD (Table I). Weight-based dosing was only required for ustekinumab—a dosage suitable for an 85 kg patient was assumed, reflecting the average weight of the Australian population.

**Table II:** Costs of best supportive care to the Australian health care system, as of September 18, 2020, and health utility inputs

Parameter	Value
Cost category	Cost (AUD)
Dermatologist visits in the induction period	113
Dermatologist visits in the maintenance period	75
Investigations in the induction period	738
Investigations in the maintenance period	80
Cyclosporine (3.0 mg/kg/day titrated up to 5.0 mg/kg/day) in the induction period	2360^15^^,^^17^
Cyclosporine (3.0 mg/kg/day titrated up to 5.0 mg/kg/day in the maintenance period	5644^15^^,^^17^
Calcipotriol monohydrate and betamethasone dipropionate foam spray in the induction period	128
Calcipotriol monohydrate and betamethasone dipropionate foam spray in the maintenance period	256
Health state	Mean QALYs (SD)
PASI 16.5 (<PASI75 response)	0.64 (0.49)^18^
PASI 4.1 (PASI75 response)	0.89 (0.11)^18^

*AUD*, Australian dollar; *PASI*, Psoriasis Area and Severity Index Score; *PASI75*, 75% improvement from the initial Psoriasis Area and Severity Index Score; *QALYs*, quality-adjusted life years*; SD*, standard deviation.

The BSC comprised cyclosporine and betamethasone dipropionate 0.05%/calcipotriol monohydrate 0.005% foam spray (Enstilar, LEO Pharma Pty Ltd) as proposed by a panel of Australian dermatologists. The mainstay of BSC was chosen to be cyclosporine, as the conventional systemic agent with the highest efficacy.^16^^,^^17^

### Utility inputs

The QALYs were accrued based on whether the patient achieved PASI75. The relevant health state utilities, which determine the number of QALYs accrued in a cycle, were derived from a preference-based utility scoring algorithm published by Matza et al,^18^ in which the utilities were calculated using time trade-off (Table II). The patient's pretreatment baseline was assumed to be PASI 16.5, the lowest value provided by Matza et al's^18^ study that was eligible for PBS subsidy under the requirement of a PASI >15 starting baseline.

### Additional analysis

An additional analysis was conducted in which the second- and third-line treatments were switched to guselkumab and ixekizumab, respectively. For patients starting on the guselkumab and ixekizumab arms, the third-line treatment was risankizumab.

### Sensitivity analyses

Sensitivity analyses were conducted to address the inherent uncertainties of economic modeling that relied on data collected from many sources, and the assumptions and input of experienced dermatologists where data was lacking.

In the one-way sensitivity analyses, the induction and maintenance period biologic efficacy, utility values of controlled and uncontrolled psoriasis, as well as costs of BSC were varied. The efficacy data were varied within confidence intervals (CIs) and in the absence of CIs, ±20% adjustments were used. This adjustment was congruent with other economic evaluations in the literature that also assessed biologics for psoriasis.^19^, ^20^, ^21^ Biologic acquisition costs were not varied as these costs were provided by the PBS and were therefore known.

In probabilistic sensitivity analyses, a Beta distribution was assigned for the utility and biologic efficacy values. A Gamma distribution was used for the BSC costs. Probabilistic ICURs were obtained from averaging the total costs and QALYs accrued after sampling from distributions of the input parameters in 10,000 iterations.

## Results

### Base-case analysis

In the base-case analysis, the treatment pathway commencing with tildrakizumab was the most cost-effective, costing AUD39,930 per patient and accruing a total of 1.57 QALYs over 96 weeks (Table III). The treatment pathway commencing with risankizumab was the most effective, accruing a total of 1.59 QALYs over 96 weeks. The treatment pathway commencing with tildrakizumab was the cheapest. The total QALYs and costs accrued for each biologic's treatment pathway in the base-case analysis are summarized in Table III.

**Table III:** The cost of and total quality-adjusted life years accrued over a 96-week time horizon for each biologic in the base-case scenario

Biologic (generic name)	Base-case efficacy (QALYs)	Base-case cost (AUD)	ICUR when compared with tildrakizumab (AUD/QALY)
Adalimumab	1.554	42,384	−172,379 (dominated)
Etanercept	1.538	45,602	−192,307 (dominated)
Guselkumab	1.584	49,720	607,746 (dominated)
Ixekizumab	1.581	48,635	674,829 (dominated)
Risankizumab	1.587	49,084	479,834 (undominated)
Secukinumab	1.582	42,696	194,524 (undominated)
Tildrakizumab	1.568	39,930	—
Ustekinumab	1.558	44,924	−492,183 (dominated)

*AUD*, Australian dollar; *ICUR*, incremental cost-utility ratio; *QALY*, quality-adjusted life year.

**Fig 2:**
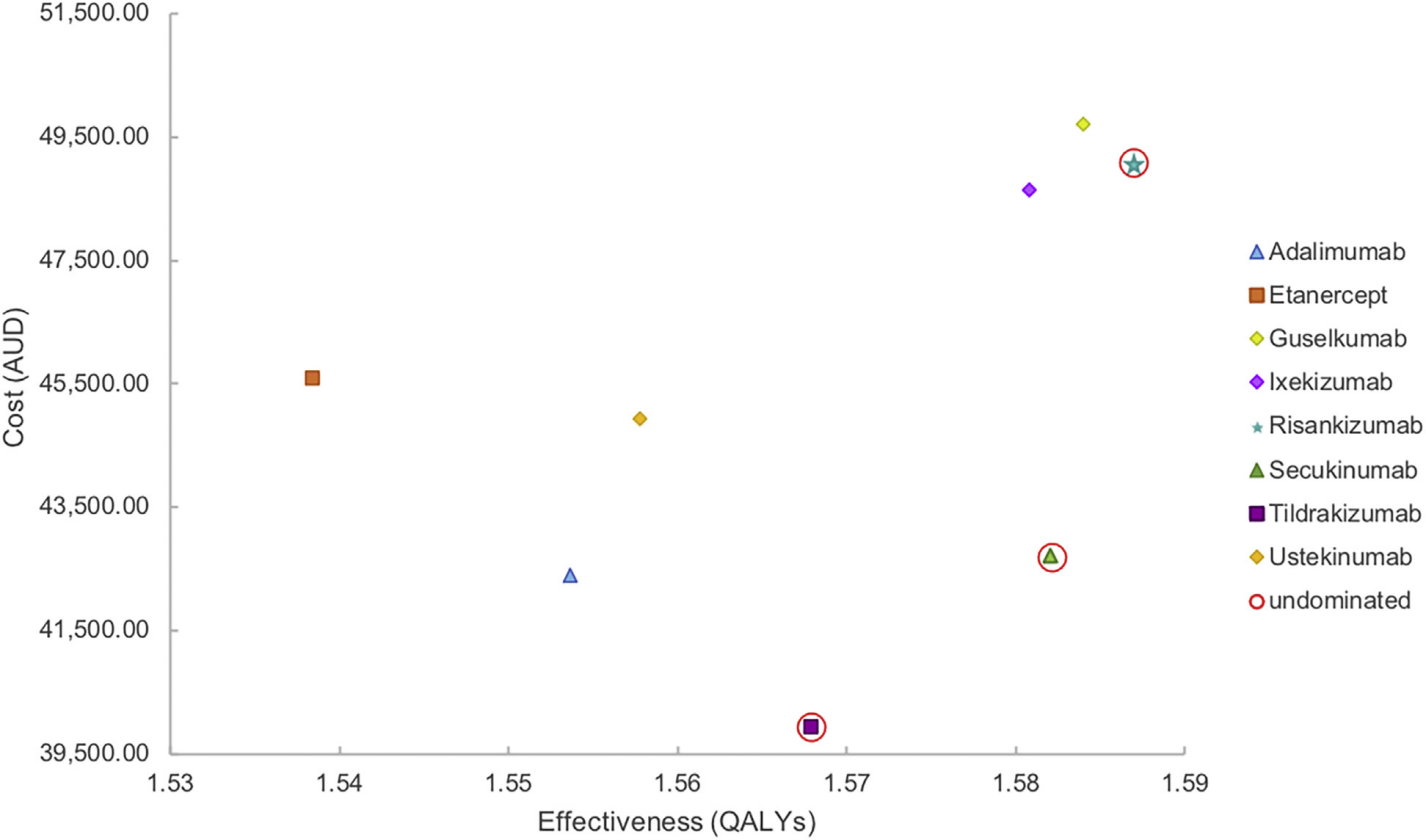
Cost-utility frontier of all Pharmaceutical Benefits Scheme-approved biologics in the base-case analysis, conducted over a 96-week time horizon. *AUD*, Australian dollar; *QALYs*, quality-adjusted life years.

As first-line secukinumab and risankizumab were more effective but more expensive than first-line tildrakizumab, they were undominated by tildrakizumab and constituted the cost-utility frontier (Fig 2). Compared with the tildrakizumab arm, secukinumab had an ICUR of AUD194,524/QALY and risankizumab had an ICUR of AUD479,834/QALY.

First-line tildrakizumab showed strong dominance over first-line ustekinumab, adalimumab, and etanercept because it was a cheaper and more effective option (Table III). First-line guselkumab had an ICUR of AUD607,746/QALY and first-line ixekizumab had an ICUR of AUD674,829/QALY when compared with first-line tildrakizumab. Because the guselkumab and ixekizumab treatment pathways had lower efficacy than that of the risankizumab treatment pathway, they were known to be extendedly dominated.

### One-way sensitivity analyses

No biologics became more cost-effective than first-line tildrakizumab when the input parameters were varied in the one-way sensitivity analyses (Supplemental Figs 2 to 8). While sensitivity analyses were conducted for all biologics as first-line treatment, the results focus on the undominated treatment strategies.

Across all one-way sensitivity analyses, first-line secukinumab's ICUR values varied from AUD77,630/QALY to AUD1,027,518/QALY when compared with first-line tildrakizumab. Secukinumab's ICUR values were the most sensitive to the variation of secukinumab's long-term efficacy within its 95% CI (ICUR range: AUD77,630-1,027,518/QALY) (Supplemental Fig 2).

Across all one-way sensitivity analyses, first-line risankizumab's ICUR values varied from AUD280,277/QALY to AUD1,666,090/QALY when compared with tildrakizumab. The ICUR was most sensitive to variation in the utility values for controlled psoriasis (ICUR range: AUD280,277-AUD1,666,090/QALY) (Supplemental Fig 3).

### Probabilistic sensitivity analyses

The probabilistic ICURs were similar to the base-case results and are summarized in Supplemental Table I. When compared with the tildrakizumab arm, first-line secukinumab and risankizumab's ICURs were AUD188,342/QALY and AUD473,386/QALY, respectively. First-line tildrakizumab dominated all other biologic options.

The cost-effectiveness acceptability curve generated from 10,000 iterations in the probabilistic sensitivity analysis (Supplemental Fig 9) shows the percentage of iterations that favor each strategy at different willingness-to-pay (WTP) thresholds. At a WTP threshold of AUD50,000, 90% of iterations favored the tildrakizumab arm, 7% favored the secukinumab arm, and 3% favored the adalimumab arm. Overall, first-line tildrakizumab was the most cost-effective pathway from the WTP thresholds of AUD0 to AUD100,000 (Supplemental Fig 9).

### Results with differing second- and third-line treatments

Additional analyses were conducted to ensure the selection of risankizumab and ixekizumab as second- and third-line treatments was not a confounder. First-line tildrakizumab remained the most cost-effective treatment pathway (Supplemental Table II).

## Discussion

The results of the base-case analysis showed that tildrakizumab was the most cost-effective first-line treatment for achieving PASI75 in adult patients with severe, chronic plaque psoriasis from the perspective of the Australian health care system. While first-line secukinumab and risankizumab were more effective than first-line tildrakizumab, their ICURs of AUD194,524/QALY and AUD479,834/QALY when compared with tildrakizumab made them highly unlikely to be cost-effective at common WTP thresholds of <AUD100,000.^22^ Tildrakizumab dominated all other biologics in the base-case analysis.

One-way sensitivity analyses showed that tildrakizumab therapy was always the more cost-effective treatment pathway, irrespective of input parameter changes. Probabilistic sensitivity analyses reinforced the cost-effectiveness of first-line tildrakizumab compared with first-line treatment with all other outpatient biologics tested. Tildrakizumab was only listed by the PBS in 2019 and approved by the US Food and Drug Administration in 2018. Therefore, its inclusion in cost-effectiveness analyses in the literature is currently limited. However, tildrakizumab was also found to be the most cost-effective biologic in a cost-effectiveness analysis conducted in England and Wales,^23^ and among the most effective in the American setting.^24^ This study's broader results suggest that the IL-23 and IL-17 inhibitors display superior cost-effectiveness compared with those of the TNF-α inhibitors and ustekinumab. These findings are consistent with other cost-effectiveness analyses conducted in the United States, Japan, Germany, Portugal, and Saudi Arabia.^19^^,^^24^, ^25^, ^26^, ^27^

In the next 4 years, the number of scripts dispensed for the use of secukinumab alone to treat chronic plaque psoriasis is expected to more than double, costing the Australian health care system in excess of AUD90 million.^28^ Given that tildrakizumab produces cost savings of AUD4,416 per year, per patient, when compared with secukinumab, the cost savings of adopting tildrakizumab as a first-line treatment could be significant.^28^

### Strengths

To our knowledge, this is the first Australian cost-utility analysis involving biologics for psoriasis. This study's adherence to PBS biologic prescription guidelines permits this cost-utility analysis to closely mimic Australian clinical practice and provide outcomes that are highly relevant for Australian dermatologists. This study also notably employed treatment sequencing in its model design, where treatment switches took place because of loss of biologic efficacy. This facilitates comparison between treatment pathways, rather than comparison between discrete treatment options, which is typical of economic evaluations accompanying RCTs.

### Limitations

In this study, the QALYs accrued for each biologic were similar (Range: 1.54-1.59 QALYs). Similar utility results between treatment arms are typical of models that employ treatment sequencing.^21^^,^^29^ However, this lends biologic acquisition cost and dosing frequency a larger role in determining cost-effectiveness. In this study, tildrakizumab had the lowest acquisition cost and frequency of dosing and was determined to be the most cost-effective drug. However, even if this study was conducted over a lifetime horizon, it is likely that the treatment savings would magnify greater than the QALY values, and it is expected that tildrakizumab would still be the most cost-effective drug. At the same time, given our shorter time horizon, this model did not incorporate drug survival rates for each biologic. A retrospective, multicenter cohort study found the cumulative probabilities of drug survival at 18 months to be in the following decreasing order: risankizumab (96.4%), guselkumab (91.1%), brodalumab (86.3%), ustekinumab (86.1%), ixekizumab (82.0%), and secukinumab (79.9%).^30^ A prospective cohort study found sustained drug survival at 1 year to be the same for ustekinumab (88%) and secukinumab (88%) but lower for adalimumab (78%).^31^ There may be a probable superiority of drug survival in IL-23 inhibitors (risankizumab and guselkumab) when compared with IL-17 inhibitors, ustekinumab, and TNF- α inhibitors, which may be of relevance for future work in this area projected to a further time horizon.^30^^,^^31^

Other limitations of this study related to the input parameters, which were chosen based on assumptions and estimations according to the best available evidence. In the absence of head-to-head comparisons of the treatment arms, evidence regarding biologic efficacy was derived from Armstrong et al's^2^ network meta-analysis. Even within Armstrong et al's^2^ meta-analysis, heterogeneity between the 60 RCTs analyzed introduced uncertainty into the results produced. Notably, Armstrong et al's^2^ network meta-analysis also identified long-term efficacy data in RCTs to be sparse. Because Armstrong et al's^2^ meta-analysis did not provide data for tildrakizumab's long-term efficacy, this data was instead obtained directly from Phase III trials.^11^ Owing to a lack of published Australian efficacy and utility data, these inputs were derived from patients participating in global RCTs and from the United Kingdom, respectively.^2^^,^^18^

Infliximab, despite being a PBS-approved biologic, was not included in this analysis because of its requirement to be administered in an inpatient setting. The biologics certolizumab and brodalumab were also excluded from this analysis as they are not currently accessible through the PBS.

Importantly, economic evaluations only provide recommendations from an economic perspective. When choosing a biologic, dermatologists must factor in other considerations, like patient comorbidities, compliance, and posology. For patients with psoriatic arthritis or coexisting cardiovascular risk factors, for example, selecting TNF-α inhibitors allows co-management of multiple disease processes.^32^ Conversely, the same TNF-α inhibitors are contraindicated in patients with a history of malignancy and multiple sclerosis.^32^^,^^33^

Finally, because of the highly specific nature of economic evaluations and this study's perspective from the Australian health care system, the application of this cost-utility analysis's results into non-Australian settings must be done with caution.

## Conclusion

When comparing the outpatient biologics available to Australian patients over a 96-week time horizon, a treatment pathway commencing on tildrakizumab is the most cost-effective. Tildrakizumab may therefore be considered as a first-line treatment for adults with severe, chronic plaque psoriasis, from the perspective of the Australian health care system.

## Conflicts of interest

Author Sebaratnam has been a consultant, speaker with honorarium, or has received travel grants from Janssen, 10.13039/501100009754Galderma, 10.13039/100006483AbbVie, 10.13039/100004319Pfizer, LEO Pharma, and 10.13039/100004336Novartis. Authors Sun, Keller, and Suresh have no conflicts of interest to declare. Sun Pharma had no role in the study design, statistical analysis, or preparation of this manuscript. Once the manuscript was finalized and accepted for publication, Sun Pharma paid the open access fee.
